# On Structural and Magnetic Properties of Substituted SmCo_5_ Materials

**DOI:** 10.3390/ma16020547

**Published:** 2023-01-05

**Authors:** Margariti Gjoka, Georgios Sempros, Stefanos Giaremis, Joseph Kioseoglou, Charalampos Sarafidis

**Affiliations:** 1 Institute of Nanoscience and Nanotechnology, National Center for Science and Research “Demokritos”, 15341 Athens, Greece; 2 Department of Physics, School of Sciences, Aristotle University of Thessaloniki, 54124 Thessaloniki, Greece

**Keywords:** Sm-Co, permanent magnet, magnetic properties, structural properties

## Abstract

SmCo_5_ is a well-established material in the permanent magnet industry, a sector which constantly gains market share due to increasing demand but also suffers from criticality of some raw materials. In this work we study the possibility of replacement of Sm with other, more abundant rare earth atoms like Ce-La. These raw materials are usually called “free” rare-earth minerals, appearing as a by-product during mining and processing of other raw materials. Samples with nominal stoichiometry Sm_1−x_MM_x_Co_5_ (*x* = 0.1–1.0) were prepared in bulk form with conventional metallurgy techniques and their basic structural and magnetic properties were examined. The materials retain the hexagonal CaCu_5_-type structure while minor fluctuations in unit cell parameters as observed with X-ray diffraction. Incorporation of Ce-La degrade intrinsic magnetic properties, Curie temperature drops from 920 K to 800 K across the series and mass magnetization from 98 Am^2^/kg to 60 Am^2^/kg; effects which trade off for the significantly reduced price. Atomistic simulations, implemented based on Density Functional Theory calculations are used in the case of the stoichiometry with x = 0.5 to calculate atomic magnetic moments and provide additional insight in the complex interactions that dominate the magnetic properties of the material.

## 1. Introduction

Many developments in modern technology are based in permanent magnets (PM), including a plentiful of critical applications in the fields of energy, automotive, robotics and more [1]. This trend is significantly enhanced in recent years, new technologies are utilized in the global fight against climate change and for productivity enhancement by introducing automation and robotics in low skill tasks. Increasing demand has pushed supply chain to its limits and fuels the urgent need for the discovery of PM based on more abundant and thus cheaper raw materials. Reducing the cost of PM can be accomplished not only by completely eliminating rare-earth raw materials, a task that has been proven quite difficult, but also by reducing the content (rare-earth lean PM) and/or by using raw rare-earth elements with larger abundance or less demand and thus less criticality and supply risk. The term Misch Metal used to refer to minerals like monazite which consist of various rare-earth compounds, mostly Ce and La; in our case we will refer strictly to Ce and La in 3:1 ratio, the symbolism MM corresponds to Ce_0.75_La_0.25_ [2,3,4,5,6].

Sm-Co system was the first high-performance RE-based PM presenting high anisotropy field (μ_0_H_A_ ~ 40 T), high Curie temperature (~1000 K) and (BH)_max_ > 150 kJ/m^3^ [7,8]. Sm is not as expensive as other rare-earth elements for the moment, however, replacing Sm with Ce_0.75_La_0.25_ in SmCo_5_ system is probably viable and has been successfully applied to other systems [9,10,11,12,13]. In the present work we have investigated the effect of introducing Ce and La in SmCo_5_ material, the structure stabilization, and the basic magnetic properties in order to investigate whether the expected cost reduction of such a material can be combined with adequately intrinsic magnetic properties. Complementary to the economic factors, our results contribute towards the better understanding of the complex nature of exchange interactions that determine the macroscopic properties and the possibility of improving the ab-initio techniques. The computational modelling and atomistic simulations already provide for better materials and thus it is of large importance to investigate and report even on less impressive compounds.

## 2. Experimental

Ingots of Sm_1−x_MM_x_Co_5_ (*x* = 0.1–1.0) samples were prepared by arc-melting constituting elements of high purity in Ar atmosphere. All raw materials are typical, commercially available products. Misch Metal consisted of 75–25 wt.% Ce-La 99 wt.%, provided by Alfa Aesar, Germany. Extra Sm was added to compensate for sublimation and all samples were melted multiple times. Materials were subsequently homogenized at temperatures 1100–1200 K for several hours in vacuum. Annealed alloys were manually crashed and ground down to less than 50 μm using a sieve of 50 mesh. Structural characterization of the powders was carried out using X-ray diffraction patterns recorded with a SIEMENS D500 diffractometer (Cu-Kα radiation). Determination of the character of the magnetocrystalline anisotropy was carried out by X-ray diffraction patterns in magnetically oriented powders which were mixed with epoxy glue and allowed to settle for adequate time within magnetic field; oriented perpendicular to the plane of X-ray incidence thus allowing only the reflections which relate to the easy magnetization direction. Magnetic measurements were carried out using a Princeton Applied Research PAR 155 vibrating sample magnetometer (VSM) at room temperature in applied fields up to 2 T while thermomagnetic analysis in low magnetic field (0.02 T) in the temperature range 300–1100 K was used to determine the critical Curie temperature.

## 3. Computational Details

Overall, Density Functional Theory (DFT) is based on the Kohn-Sham theory [14], which effectively maps the 3N-dimensional problem of explicitly solving the Schrodinger equation for an N-body system of interacting electrons to the treatment of a system of N coupled, 3 dimensional equations, describing a system of non-interacting electrons with the same density as the original one [15]. An integral development towards the Kohn-Sham theory were the Hohenberg-Kohn theorems, which shown that the external potential (and consequently the groundstate Hamiltonian) of a system of electrons can be uniquely expressed as a functional of the electron density, and that the groundstate electron density minimizes the functional of the groundstate energy [16]. The proof of the first part was performed by a reductio ad absurdum argument by E. S. Kryachko [17], who provided an alternative derivation of the first Hohenberg-Kohn theorem. The accuracy of DFT in describing exchange coupling has been studied extensively and has been found that the choice of functional has a substantial impact (e.g., considering the broken symmetry density functional theory (BS-DFT) approximation [18]).

The DFT calculations were performed within the spin polarized and rotationally invariant DFT+U framework by Dudarev et al. [19] and the the Perdew-Burke-Ernzerhof derivation of the gene-ralized gradient approximation pseudo-pontentials [20], with the projector augmented-wave method [21] by using the VASP code [22]. The value of the +U potentials was set to 4.7 eV for the 4f electrons of Sm and Ce and 2.22 eV for the d electrons of Co and La, in agreement with the literature [23]. Calculations were performed on a dataset of 96 atom, 2 × 2 × 4 supercell configurations with the MM_0.5_Sm_0.5_Co_5_ stoichiometry, created with the SUPERCELL code [24]. In this approach, the La and Ce atoms substitute half of the Sm ones in a pseudorandom manner. The structural, electronic, and magnetic properties were extracted from the energetically favourable configuration. The visualization of the atomically resolved DOS was performed with the PyProcar code [25].

## 4. Results and Discussion

### 4.1. Structural Characterization

**Figure 1:**
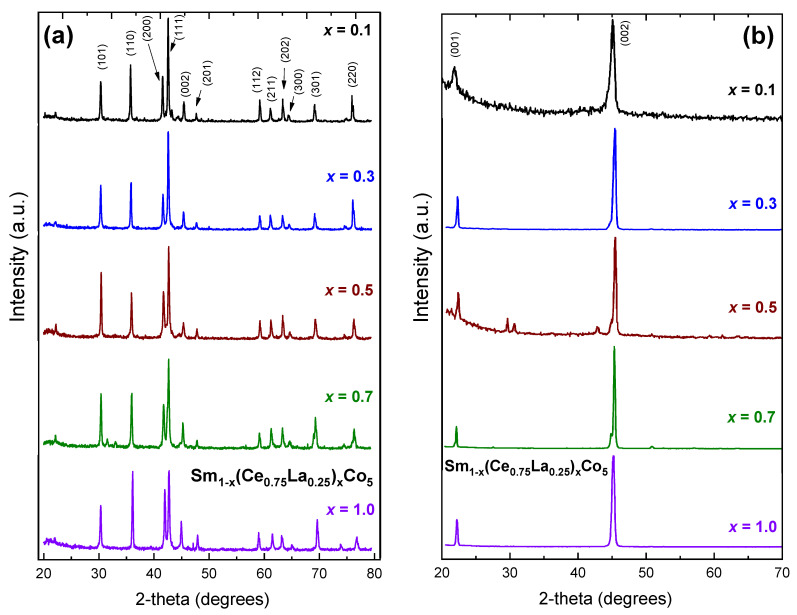
X-ray diffraction plots (Cu Κα radiation) of Sm_1−x_(Ce_0.75_La_0.25_)_x_Co_5_ (x = 0.1–1.0) compounds; (**a**) powder samples; (**b**) magnetically oriented samples.

**Figure 2:**
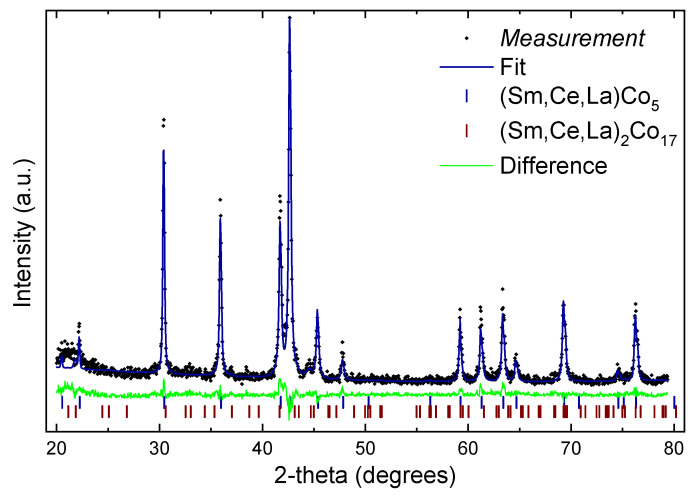
Rietveld analysis of Sm_1−x_(Ce_0.75_La_0.25_)_x_Co_5_ (x = 0.5) compound.

In Figure 1 X-ray diffraction plots of the materials under study are presented. All samples present the typical hexagonal CaCu_5_-type signature pattern of the SmCo_5_ system (S.G. No 191, P6/mmm). Small amount of the hexagonal Th_2_Ni_17_-type phase (S.G. No 194, P6_3_/mmc) was detected, in most cases up to 5 wt.% and close to 10 wt.%. for x > 0.5. Relative intensity of some reflections is not retained for higher Ce, La Misch Metal content and the quality of the Rietveld analysis is degraded, indicating either a possible preference for the remaining Sm atoms or the introduction of strains in the structure. In all samples we have fixed the rare-earth atomic populations assuming the nominal composition. The stabilization of the hexagonal structure is possible within all the stoichiometry range. In Table 1 the unit cell parameters of the materials are summarized, and they are plotted against Ce_0.75_La_0.25_ content in Figure 2.

In Figure 1b X-ray diffraction plots the uniaxial character of all compounds is evident, as expected [26,27,28]. In all cases the (001) and (002) reflections of the CaCu_5_-type structure are visible, indicating easy-axis type of anisotropy perpendicular to the basal plane of the hexagonal structure. In the case of the sample with x = 0.5 some additional, minor peaks are observed, at two-theta angles of 29.7, 30.7 and 42.8 degrees. The two latter correspond to strong reflections of the P6/mmm hexagonal structure, namely (011) and (111), indicating the possibility of misalignment in the sample. However, the additional peak at 29.7 degrees two theta does not match with any significant reflection of the CaCu_5_-type, Th_2_Ni_17_-type or Th_2_Zn_17_-type structures. Therefore, we have to consider the possibility of existence of an unknown minor phase, not possibly detectable in the random powder X-ray diffraction patterns.

In Figure 2, a plot of the sample with x = 0.5 combined with a theoretical fit using Rietveld analysis is provided. The amount of the secondary, Th_2_Ni_17_-type phase (S.G. No 194, P6_3_/mmc) is about 9 wt.%. In all cases the amount of secondary phase was between 3 wt.% (x = 0.1) and 10 wt.% (x = 0.7) while the quality of the fit in all cases was about 2–3% higher than R_exp_, for example in the case of x = 0.5, R_p_ = 19%, R_exp_ = 16%. We have examined the possibility of existence of both main variants of 2:17 phases, Th_2_Ni_17_-type and the rhombohedral Th_2_Zn_17_-type (S.G. No 166, R −3 *m*) and a combination of both. Due to the small amount of the secondary phase, there is no clear result. It is known that Sm_2_Co_17_ usually crystallizes in the rhombohedral variant and Ce_2_Co_17_, La_2_Co_17_ mostly in the hexagonal [29,30]; both have the same basal plane, the same orientation of the c-axis and occur from 1:5 with the same amount of replacement of rare-earth atoms with transition metal pairs, their difference is due to the stacking sequence [31].

**Figure 3:**
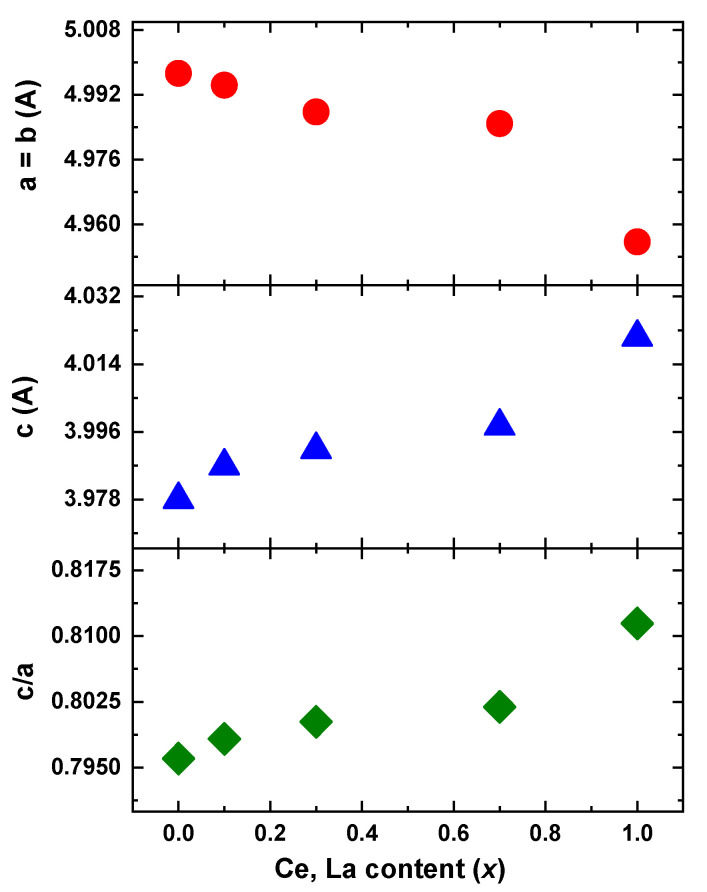
Unit Cell parameters for Sm_1−x_(Ce_0.75_La_0.25_)_x_Co_5_ (x = 0.1–1.0) compounds.

From the values of the table and the Figure 3 some clear trends are observed. The introduction of Ce, La decreases the basal plane unit cell parameters while it increases the perpendicular c-axis. The c/a ratio increases as well. It is well known [31,32,33] that the hexagonal RCo_5_ compound crystallizes in a layered structure, where Co only Kagome layers alternate with mixed R-Co layers. The hexagonal Th_2_Ni_17_-type phase is very related to the basic structure and is produced by the partial replacement of one third of rare earth atoms with pairs of transition metal, aligned parallel to the c-axis of the structure and called dumbbells. Slightly disordered variations or other impurities are also very common in the system [34]. The incorporation of different rare earth atoms, as in our case, may enhance such effects. The small variations in peak intensities in Figure 1 may relate to such structural and compositional discrepancies. The magnetic properties are obviously sensitive to these parameters but the close structural relation of them is not an obstacle from the applications and industrial manufacturing/processing scope.

### 4.2. Ab Initio Electronic and Magnetic Properties for x = 0.5

**Figure 4:**
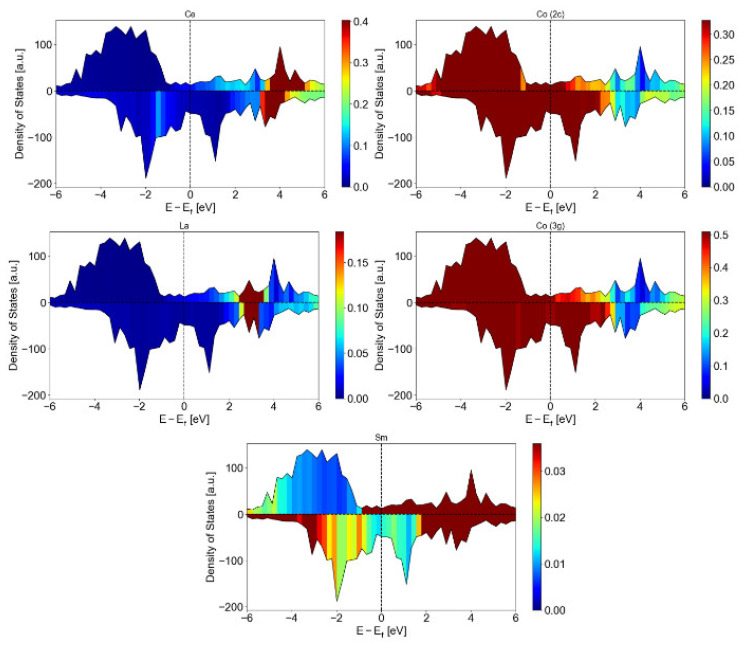
Atomic contribution to the electronic DOS for RE atoms (Ce, La, Sm) and Co (2c and 3g sites) as calculated from the ab initio simulations.

The results of DFT calculations for the atomically resolved, electronic DOS for a Ce_0.75_La_0.25_ content of x = 0.5 in the energetically preferable structure are presented in Figure 4. The contribution of the Co sublattice to the electronic states for both the 2c and the 3g sites dominates a broad range close to the Fermi level and pronounces its importance for the magnetic properties of the RE-Co_5_ structure. The peaks associated to the Ce atoms are close to 4 eV, while the ones associated to the La atoms are close to 3 eV. The contribution of Sm atoms is found to less pronounced and over a broader range above the Fermi level. The respective average magnetic moments for each atom type are also presented in Table 2.

The treatment of the strongly correlated 4f electrons of Sm within DFT is a known challenge. In this work, we employed the implementation so-called standard rare earth model for Sm within VASP and the PAW-PBE-GGA pseudopotentials, which treats the 4f valence electrons of Sm as core states. The SRM was previously demonstrated for SmCo_5_ to agree with the dynamical mean field calculations with the Hubbard I solver (DMFT–H1A), using the full-potential linear muffin-tin orbital method (FPLMTO), which were claimed to provide the most accurate description for SmCo_5_, but at an elevated computational cost [35,36], and provide reasonable results for the whole lanthanide series [37]. The explicit treatment of 4f electrons as valence in the planewave PAW formalism is known to lead to self-interaction errors and a spurious pinning of the 4f orbitals of Sm to the Fermi level, and, in turn, demonstrated significantly overestimate the local magnetic moments of Sm atoms [38,39]. The aim of the ab initio simulations in this work was to investigate the local atomic magnetic moments of the system. Therefore, by using PAW-GGA with the SRM as implemented in VASP, in combination with the appropriately parametrized +U approach, we were able to obtain reasonable results for the local atomic magnetic moments and the total magnetization, compared to experimental values.

However, with this approach, we found an overly pronounced value of magnetocrystalline anisotropy energy (MAE) for SmCo_5_ (~37 meV/f.u., compared to 10.5 meV/f.u. from SRM-FPMLTO calculations of [36], experimental values ranging between 9–16 meV [40,41,42,43,44,45]). In an older study, Larson et al. employed GGA+U calculations within the full-potential, relativistic linearized augmented plane wave (FLAPW) approach with U = 5.2 eV and J = 0.75 eV and found a MAE of 12.6 and 21.6 eV (depending on the approach involved in the implementation of the Hubbard correction in WIEN2K.01 and WIEN2K.02 packages) but their calculated magnetic moments had a discrepancy of 2–3 μB compared to experimental values [46]. They also mention that a lower value of U close to 4 eV leads to a significant overestimation of the MAE at ~40 meV/f.u., which agrees with our results (U = 4.7 eV, MAE = 37 meV/f.u.). Therefore, in our approach, we used a value of U which resulted to a more accurate description of the magnetic moments, which was our main point of interest, at the cost of an overestimated MAE, and we did not investigate the impact of substituting Sm to the latter quantity.

### 4.3. Magnetic Properties

**Figure 5:**
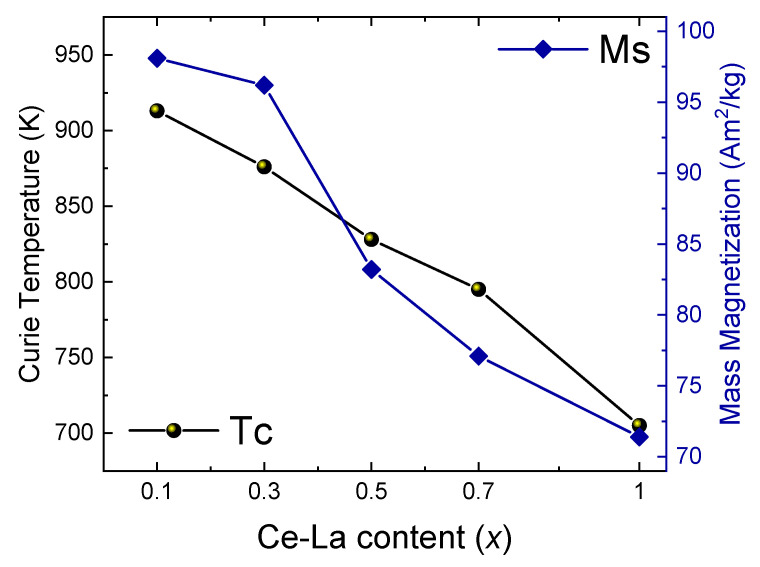
Dependence of magnetic properties with Ce,La content in Sm_1−x_(Ce_0.75_La_0.25_)_x_Co_5_ (x = 0.1–1.0) samples.

Thermomagnetic analysis under small, constant magnetic field was used to determine critical transition temperatures (Table 3, Figure 5). For all stoichiometry range a drop in the Curie temperature is observed, almost linear with Ce-La content. Mass magnetization follows the same trend as Curie temperature, reflecting the overall weakening of the magnetic interactions (Figure 4). It is interesting to observe that ab-initio calculations for the sample with x = 0.5 present a larger magnetization compared to the experimental evaluation. Imperfections and minor impurities may account for that difference. The structural effects we have already described are an additional cause for this weakening of the magnetic properties. For low Ce, La Misch Metal content the degradation of the intrinsic magnetic properties is rather small, thus it is reasonable to consider the possibility of using Ce/La dopped SmCo_5_ as a base material for preparing permanent magnets due to the reduced cost. Chen et al. [19] have demonstrated that such doped Sm-Co / a-Fe nanocomposites present adequate remanence and coercivity and in addition to the other commercial and production factors which have been already discussed here are an interesting choice for PM optimization. We must also take into account the fact that Ce and La are the rare earths with the largest production volume and are considered as oversupplied raw materials [47]. Their large availability and lower price may be considered for confronting the balance problem in the rare earth raw materials supply.

## 5. Conclusions

Single phase samples of nominal stoichiometry Sm_1−x_(Ce_0.75_La_0.25_)_x_Co_5_ (x = 0.1–1.0) were successfully synthesized by conventional metallurgy methods. The hexagonal structure of the mother SmCo_5_ compound is formed for all stoichiometry range, together with the crystallographically similar Th_2_Ni_17_-type compound for high Ce, La content. Main intrinsic magnetic properties, Curie temperature and mass magnetization, are weakening, especially for higher Ce, La replacement for Sm, more than 30%. This effect is minimal for lower Ce-La content; considering the low cost and the availability of the specific minerals we conclude that the possibility of introducing Ce, La Misch Metal in commercial SmCo_5_ permanent magnets is feasible. The dependence and interconnection of the magnetic properties on the structural parameters provide an insight for further research, the adjustment of the observed trend in this work with additional substitutions, like partial substitution of Co with other transition metals, may compensate for the effect and provide a better material with the additional reduction of Co content; since Co is an even more critical resource this path could be proven important in the future.

## Figures and Tables

**Table 1:** Unit Cell Parameters for Sm_1−x_(Ce_0.75_La_0.25_)_x_Co_5_ (x = 0.1–1.0) main phase.

x	a = b (Å)	c (Å)	c/a
0.1	4.9973(10)	3.9780(9)	0.7960
0.3	4.9944(7)	3.9869(6)	0.7983
0.5	4.9878(14)	3.9913(12)	0.8002
0.7	4.9849(15)	3.9975(13)	0.8019
1.0	4.9557(6)	4.0212(8)	0.8114

**Table 2:** Average magnetic moments for each atomic species as calculated from the ab initio simulations for the case of the sample with x = 0.5.

Atomic Average	m/atom (μ_B_/atom)
Sm	−0.33
La	−0.32
Ce	−1.29
Co (2c)	1.64
Co (3g)	1.63

**Table 3:** Curie temperature and magnetization values of Sm_1−x_(Ce_0.75_La_0.25_)_x_Co_5_ (x = 0.1–1.0) samples. Reference undoped SmCo_5_ value from [48].

x	T_c_ (K)	M_S_ (Am^2^/kg)	M_S_ (μ_Β_/f.u.)
0	1020	102	8.15 [48]
0.1	913	98.1	7.80
0.3	876	96.2	7.61
0.5	828	83.2	6.55
0.7	795	77.1	6.10
1.0	705	71.4	5.91

## Data Availability

Data not already included in the article are available on request from the corresponding author.
